# Diagnostic and prognostic potential of biomarkers in femoroacetabular impingement syndrome: A systematic review

**DOI:** 10.1002/jeo2.70417

**Published:** 2025-09-22

**Authors:** Junya Yoshitani, Seper Ekhtiari, Joseph Dulleston, Ajay Malviya, Vikas Khanduja

**Affiliations:** ^1^ Young Adult Hip Service, Department of Trauma and Orthopaedics Addenbrooke's ‐ Cambridge University Hospitals NHS Foundation Trust Cambridge UK; ^2^ School of Clinical Medicine University of Cambridge Cambridge UK; ^3^ Northumbria Hip Preservation Unit, Department of Trauma and Orthopaedic Surgery Northumbria Healthcare NHS Foundation Trust Cambridge UK; ^4^ Department of Surgery, Division of Trauma and Orthopaedics University of Cambridge Cambridge UK

**Keywords:** biomarkers, diagnosis, disease progression, femoroacetabular impingement syndrome, hip osteoarthritis

## Abstract

**Purpose:**

Early diagnosis of femoroacetabular impingement syndrome (FAIS) is essential. This systematic review aimed to identify biomarkers useful for diagnosing FAIS and predicting its progression to hip osteoarthritis. Our hypothesis was that there are biomarkers that are useful for the diagnosis and/or prognosis of FAIS. Our research questions were: (1) which biomarkers support diagnosis or screening of FAIS? and (2) which biomarkers predict disease progression?

**Methods:**

A systematic review using the PRISMA guidelines was conducted to investigate the relationship between biomarkers and FAIS. The diagnosis of FAIS was based on the criteria used in each original study, typically involving clinical symptoms and radiographic evidence of CAM or pincer morphology. The protocol for the review has been published in PROSPERO. Literature search was performed using three databases: Embase, MEDLINE and Cochrane Library. The initial search yielded 683 articles of which 16 articles were included for final analysis. Data from a total of 2134 participants were analysed. Sixty‐eight unique biomarkers associated with FAIS were identified and measured.

**Results:**

Diagnostically, 19 biomarkers were identified, of which 12 could significantly detect a difference between patients with FAIS and healthy controls. Forty‐two biomarkers predicting the association of FAIS with hip osteoarthritis or late FAIS were identified, of which 16 biomarkers were statistically significant. Only 4‐aminobutyrate aminotransferase promoter (ABAT) and peroxisome proliferator‐activated receptor gamma (PPARγ) were associated with both diagnosis and prognosis.

**Conclusions:**

Biomarkers may support the diagnosis and monitoring disease progression in patients with FAIS. Twelve biomarkers may detect early changes, and 16 may predict progression to osteoarthritis. Further refinement is required to identify those most useful in clinical practice. ABAT and PPARγ may be linked to both diagnosis and progression. While primarily preclinical, these findings may improve diagnostic accuracy, reduce overtreatment and aid decisions regarding joint preservation strategies.

**Level of Evidence:**

Level III.

**Abbreviations**
ABAT: 4‐aminobutyrate aminotransferase promoter
ADAMTS‐4: a disintegrin and metalloproteinase with thrombospondin motifs 4
ALP: alkaline phosphatase
COL10A1: collagen type X alpha 1 chain
COL2A1: collagen, type 2, alpha
COMP: cartilage oligomeric matrix protein
CRP: C‐reactive protein
CTX‐I: C‐terminal telopeptide of collagen type I
DNMT1: DNA methyltransferases 1
ELISA: enzyme‐linked immunosorbent assay
FAIS: femoroacetabular impingement syndrome
HA: hyaluronic acid
IL‐1β: interleukin‐1β
IL‐6: interleukin‐6
JBI: Joanna Briggs Institute
MMP‐13: matrix metalloproteinase 13
MRI: magnetic resonance imaging
NITEGE: aggrecan neopeptide
NTX‐I: N‐terminal telopeptide of collagen type I
OA: osteoarthritis
OC: osteocalcin
OPG: osteoprotegerin
PINP: N‐terminal propeptide of procollagen type I
PPARγ: peroxisome proliferator‐activated receptor‐gamma
PRISMA: preferred reporting items for systematic reviews and meta‐analyses
RANKL: receptor activator of nuclear factor‐kB ligand
RT‐PCR: reverse transcription polymerase chain reaction
TNFα: tumour necrosis factor‐alpha
VEGF: vascular endothelial growth factor

## INTRODUCTION

Femoroacetabular impingement syndrome (FAIS) is a common cause of hip pain and reduced hip range of motion in young adults [10, 17, 37]. In about 53%–81% of patients, FAI can progress to hip osteoarthritis (OA) [2, 17, 45]. Early diagnosis is therefore critical to the success of joint‐preserving procedures, which can prevent further cartilage degeneration and reduce the risk of developing hip OA [6, 20, 34, 35, 41]. Despite significant advances in imaging techniques including magnetic resonance imaging (MRI), identification and accurate preoperative quantification of articular cartilage degeneration in the hip in the early stages remains a challenging proposition [8].

Recently, molecular biomarkers have gained popularity as a useful adjunct in diagnosing musculoskeletal conditions such as periprosthetic joint infection and hip OA [5, 11, 25, 38, 43]. Although molecular biomarkers have the potential to diagnose FAIS and predict progression to hip OA [38, 48], aetiological uncertainty contributes to the complexity and challenge of developing and using molecular biomarkers [33, 38, 50]. Moreover, given that the degree of cartilage damage is linked to a higher likelihood of unfavourable surgical results [14, 22, 24, 32, 36], molecular biomarkers may help to redefine indications for joint preserving surgery and facilitate more timely intervention [26, 51].

Although a previous systematic review published in 2019 comprehensively assessed 43 biomarkers in FAIS and hip OA [33], it did not stratify the diagnostic and prognostic utility based on control groups such as healthy individuals or OA patients. Furthermore, while the previous review discussed the potential diagnostic and prognostic value of certain biomarkers, such as COMP and FAC, it did not provide a comprehensive, stratified assessment of their clinical utility. In addition, several novel biomarkers have been investigated since then [21, 26, 40]. It remains unclear to what extent previously reported biomarkers are clinically applicable in diagnosing FAIS or in predicting its progression to hip OA. Therefore, this systematic review aimed to comprehensively identify and categorise biomarkers associated with FAIS, particularly focusing on their clinical relevance in diagnosis and in predicting progression to OA.

The purpose of this review was to summarise and evaluate biomarkers related to the pathophysiology of FAIS. Our hypothesis was that there are biomarkers that are useful for the diagnosis or prognosis of FAIS. Therefore, our specific research questions were: (1) Are there reliable and specific biomarkers for diagnosing FAIS? and (2) Do any of these biomarkers have the potential to predict progression to hip OA?

## METHODS

### Protocol and registration

This study was conducted according to the Cochrane Handbook for Systematic Reviews reported as per the preferred reporting items for systematic reviews and meta‐analyses 2020 (PRISMA) guidelines [39] and registered in PROSPERO.

### Eligibility criteria

Studies which assessed the pathology of FAIS by measuring biomarkers were investigated. The eligibility criteria for the literature review were determined before the search by the authors. The inclusion criteria for this systematic review were as follows: (1) studies on all human subjects (all ages and both sexes); (2) studies involving biomarkers; (3) assessment of patients with FAIS or strongly suspected of having FAIS; and (4) English language studies. The diagnosis of FAIS was based on the criteria used in each original study, typically involving clinical symptoms and radiographic evidence of cam or pincer morphology. The exclusion criteria were as follows: (1) Publications that were not accessible in full text (e.g., conference abstracts or papers available only as abstracts); (2) studies that were not original (e.g. systematic reviews, narrative reviews, editorial commentaries and technical notes); (3) studies in which FAIS could not be clearly identified as the condition under investigation; (4) studies that exclusively assessed imaging modalities, as these are not molecular biomarkers and fell outside the scope of this review and (5) Studies that included patients with dysplasia, previously undergone operative treatment or other hip pathologies were excluded.

### Information sources and search strategy

The review included original data articles that were retrieved through computer‐assisted searches of Embase, MEDLINE and the Cochrane Library. Prior to final analysis, additional searches were conducted to identify any recent studies for possible inclusion. The search was conducted by two reviewers (Author 1, Author 2) on 24 March 2023, using following MeSH terms and keywords with the help of Boolean operators. ‘biomarker’, ‘cam morphology’, ‘cam deformity’, ‘pincer morphology’, ‘pincer deformity’, ‘femoroacetabular impingement’, ‘femoroacetabular‐impingement’, ‘FAI’ and ‘hip osteoarthritis’. A detailed explanation of our search strategy is provided in the Supplementary Material S1.

### Selection process

Results of electronic database searching were imported into Endnote 20 (Clarivate Analytics) and Rayyan (Rayyan Systems) and deduplicated. Study titles and abstracts were screened by two independent reviewers (Author 1, Author 2) to determine whether they meet our inclusion criteria. Any disagreements were settled through discussion with a third reviewer and senior author (Author 5). Full texts were then read to determine the final studies to be included in our qualitative synthesis. If the two initial reviewers (Author 1, Author 2) didn't reach a consensus, they consulted a third, more senior reviewer (Author 5).

### Data collection process

A data extraction form was developed in Excel (Microsoft) and piloted. Data was extracted by two independent reviewers (Author 1, Author 2). Any discrepancies were settled in a consensus meeting and in discussion with a third reviewer (Author 5).The following data were extracted: first author, year of publication, study design, number of participants, participant demographics, the type and source of biomarkers, assay methods used and statistical analysis of biomarker levels in various patient groups. When available, diagnostic criteria, alpha angle, centre‐edge angle (CEA) and grade of osteoarthritis (e.g., Tönnis grade) were also noted. Diagnostic information and definitions used in each study are summarised in Supplementary Material S2. The primary outcome of this review was to identify and categorise biomarkers reported in the literature that are significantly associated with the diagnosis of FAIS or the prediction of its progression to hip osteoarthritis.

### Risk of bias assessment

Methodological quality was critically assessed for each of the included studies using the Joanna Briggs Institute (JBI) critical appraisal tools, including the cross‐sectional studies critical appraisal checklist (Supplementary Material S2) [4]. One reviewer (Author 1) read the included publications and answered all the questions on the quality appraisal checklists. A second reviewer (Author 2) checked all the quality appraisal results. Discrepancies were discussed with the review team if required until consensus was achieved. Two reviewers independently extracted relevant study data from the final pool of included articles and recorded these data on an Excel spreadsheet.

### Synthesis method

Data was synthesised by two reviewers (Author 1, Author 2) without blinding. Biomarkers were categorised according to Lotz et al. [31], based on their nature and molecular origin, into four classes: (1) biomarkers associated with the metabolism of collagen in cartilage (type II collagen) or subchondral bone (type I collagen); (2) biomarkers associated with the metabolism of aggrecan in cartilage; (3) biomarkers related to a range of noncollagenous proteins that have a role in other metabolic pathways in the joint, including glycoproteins, proteoglycans, metalloproteinases and advanced glycation end‐products, as well as hyaluronan, which is a constituent of both cartilage and synovium and (4) biomarkers associated with other processes, such as inflammation or fibrosis [9]. Biomarkers were categorised based on their clinical relevance in diagnosing FAIS and predicting its progression to hip OA. To achieve this, biomarkers were grouped according to the type of control group used in each study: (1) healthy individuals (diagnosis group), (2) OA or late‐stage FAIS patients (disease progression group) and (3) other specific or nonstandard comparators (other group). The ‘other group’ refers to studies in which biomarkers were compared between nonconventional groups, such as pre‐ and postoperative values within the same FAIS cohort or comparisons that did not involve either healthy or OA controls. For diagnosis group and disease progression group, biomarkers were classified into five groups; (1) biomarkers significantly related to both ‘diagnosis’ and ‘disease progression’, (2) biomarkers significantly related to ‘diagnosis’, (3) biomarkers significantly related to ‘disease progression’, (4) No significant relation and (5) Opinions were divided. Given the qualitative and heterogeneous nature of the included studies—covering various biomarker types, measurement techniques and outcome definitions—no quantitative synthesis or statistical analysis was performed. Therefore, meta‐analysis or pooled statistics were not applicable. Instead, data were synthesised qualitatively and classified according to relevance and level of evidence, as described in the synthesis method above.

## RESULTS

### Search results

**Figure 1:**
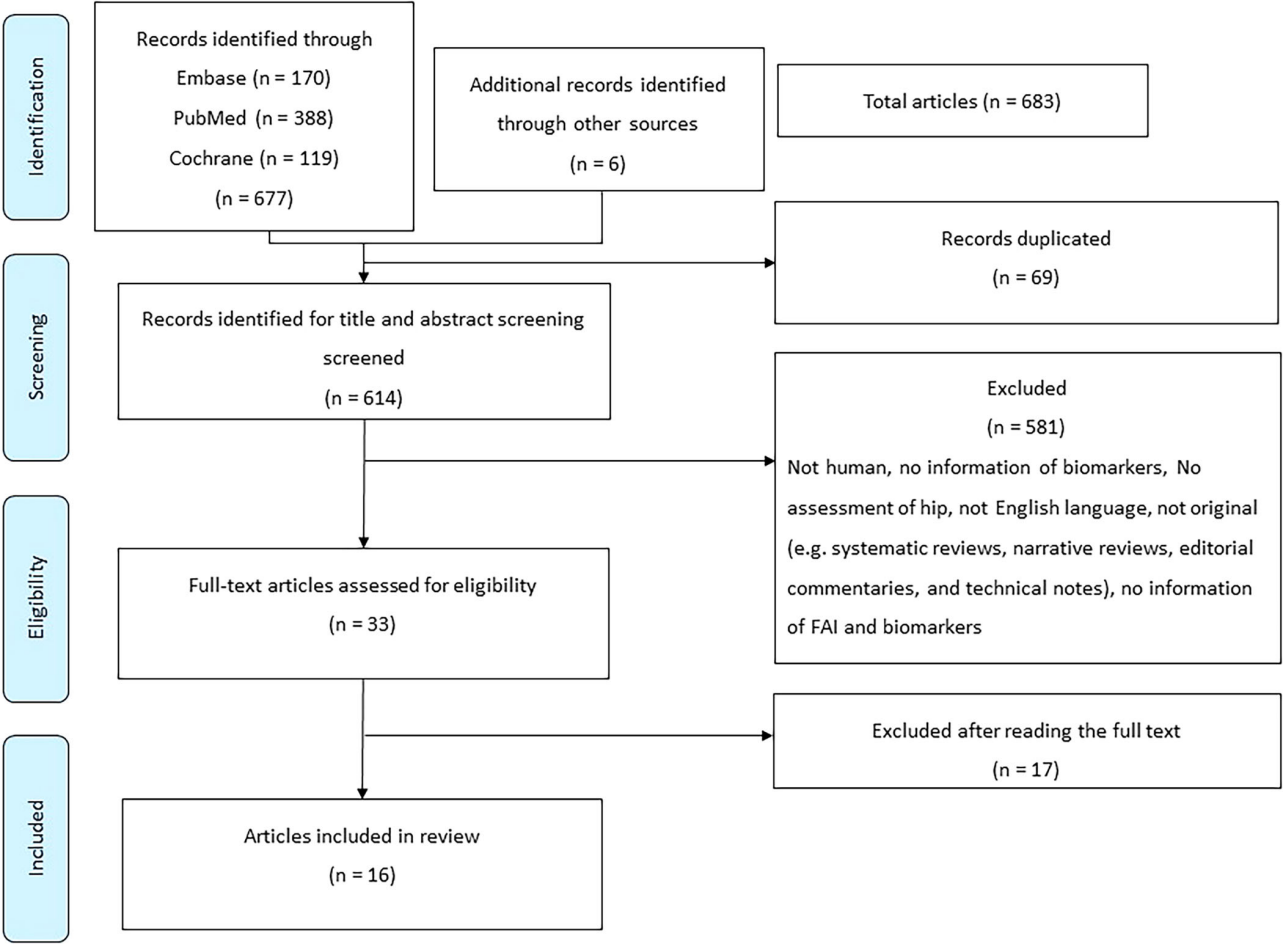
Flow diagram of included and excluded publications.

Six hundred and seventy‐seven reports were identified via electronic database searching, with six further reports were gleaned from the ascendency approach (Figure 1). 614 reports remained after deduplication (Figure 1). After the exclusion of 581 studies following title and abstract screening, 33 full texts were deemed relevant. Assessment of these texts yielded 18 final studies. Two studies were deleted because each study was suspected to represent the same cohort of patients, which left 16 studies for final analysis (Figure 1).

### Patient characteristics and quality of included studies

2134 participants were identified (mean age 42.8 years, 1531 female, 583 male, 20 unknown gender) (Table 1). Thirteen studies include FAIS patients diagnosed by clinical examination and imaging; one study was a large cohort with knee and hip pain including information about cam deformity; patients of one study included primary OA which was suspected to be secondary to FAIS based on images in the published manuscript; and one study included patients undergoing hip arthroscopy for FAIS or labral pathology but without OA. Further patient details about the diagnosis and control groups are shown in Supplementary Material S2.

**Table 1:** Summary of the study characteristics.

Author	Year	Location	Study design	Number of participants	Male	Female	MA	Data sources	Tissue examination
van Spil et al.	2015	Netherlands	Prospective, cross‐sectional study	1583	325	1258	55.8	Serum, plasma, and second‐morning void urine samples	ELISA or radioactive immunoassay
Shapiro et al.	2016	USA	Prospective, cross‐sectional study	16	4	12	38.9	Synovial fluid samples	ELISA immunofluorescence
Haneda et al.	2020	USA	Retrospective, cross‐sectional study	30	24	6	32.5	Synovial fluid samples	Immunostaining
Bedi et al.	2013	USA	Prospective, cross‐sectional study	29	29	0	22.6	Plasma samples	ELISA
Talks et al.	2019	UK	RCT	115	38	77	37	Serum samples	Immunoturbidimetry
Kamenaga et al.	2023	USA	Prospective, cross‐sectional study	29	15	14	40	Cartilage samples	Immunofluorescence, western blots, PCR, histological
Kuhns et al.	2022	USA	Retrospective, cross‐sectional study	37	22	15	46.5	Cartilage samples	whole genome RNA sequencing RT‐PCR immunohistochemical
Turdean et al.	2017	Romania	A prospective observational study	57	32	25	67.69	Synovial fluid samples, synovial membranes	Immunostaining
Elias Jones et al.	2015	UK	Retrospective, cross‐sectional study	20			37.5	Labrum from impingement zone of the lesion	Histology and immunohistochemistry
Abrams et al.	2017	USA	Prospective, cross‐sectional study	17	9	8	33.4	Synovium	Immunofluorescence Analysis
Abrams et al.	2014	USA	Prospective, cross‐sectional study	34	14	20	48.8	Synovial fluid samples	immunofluorescence
Fukushima et al.	2017	Japan	Prospective, cross‐sectional study	33	10	23	41.8	Synovial membranes samples	RT‐PCR
Hashimoto et al.	2013	USA	Prospective, cross‐sectional study	35	22	13	30.1	Articular cartilage samples	RT‐PCR
Pascual‐Garrido et al.	2022	USA	Retrospective, cross‐sectional study	21	13	8	55.5	Articular cartilage samples	RT‐PCR
Gao et al.	2021	China	Retrospective, cross‐sectional study	18	6	12	41	Bone tissue samples from head‐neck junction	RT‐PCR
Chinzei et al.	2016	Japan	Retrospective, cross‐sectional study	60	20	40	55.5	Cartilage, synovium and labrum	RT‐PCR

Abbreviations: ELISA, enzyme‐linked immunosorbent assay; MA, mean age; RT‐PCR, reverse transcription polymerase chain reaction.

### Study quality assessment

The JBI's analytical cross‐sectional studies critical appraisal tool was used for all studies [4]. Most of the critical appraisal checklist items were confirmed as ‘yes,’ indicating acceptable methodological quality across studies. However, the strategy to address confounding factors was marked as ‘no’ in all but two studies, largely due to the inherent limitations in sample collection and study design in basic science research. No studies were excluded based on the quality assessment, which was conducted for informational purposes only. Overall, while the studies were generally of moderate quality, limitations related to potential confounding and lack of standardised reporting should be considered when interpreting the results. Detailed results of the quality assessment are provided in Supplementary Material S3.

### Overview of biomarkers

A total of 68 unique biomarkers were identified across 16 studies (Supplementary Material S4). The most frequently evaluated markers were matrix metalloproteinase 13 (MMP‐13), interleukin‐1β (IL‐1β), and metalloproteinase with thrombospondin motifs 4 (ADAMTS‐4). Samples were derived from cartilage, bone, synovial fluid, serum and labrum tissues, and analysed using the enzyme‐linked immunosorbent assay (ELISA), reverse transcription polymerase chain reaction (RT‐PCR) and other standard assays.

### Diagnostic and prognostic utility

**Figure 2:**
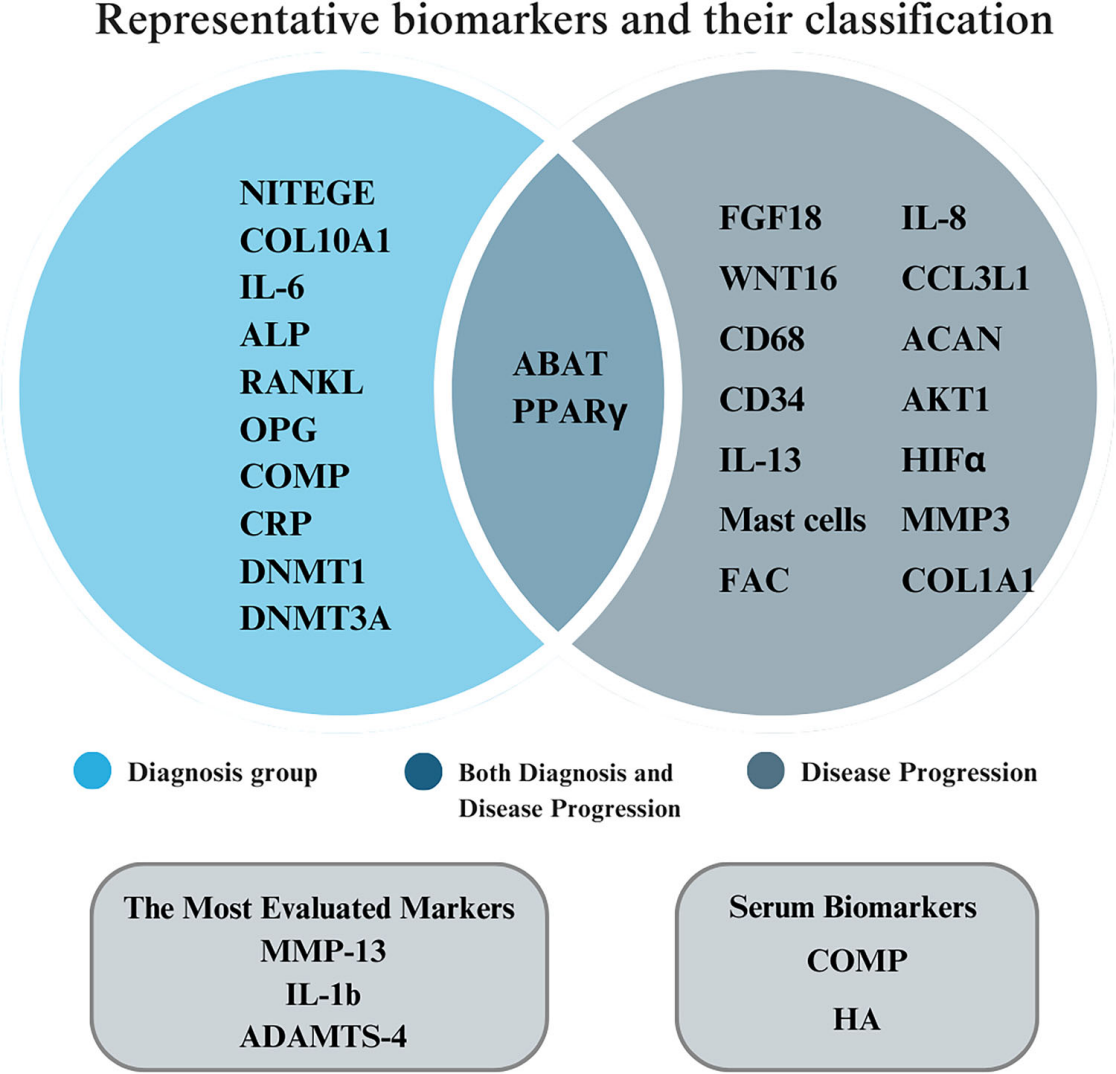
Representative biomarkers and their classification in this review. ABAT, 4‐aminobutyrate aminotransferase promoter; ACAN, aggrecan; ADAMTS‐4, A disintegrin and metalloproteinase with thrombospondin motifs 4; ALP, alkaline phosphatase; CCL3L1, chemokine (C‐C) motif ligand 3‐like 1; CD, cluster of differentiation; COL1A1, collagen, type 1 alpha 1; COL10A1, collagen type X alpha 1 Chain; COMP, cartilage oligomeric matrix protein; CRP, C‐reactive protein; DNMT, DNA methyltransferases; FAC, fibronectin–aggrecan complex; FGF18, fibroblast growth factor 18; HA, hyaluronic acid; IL‐1β, interleukin‐1 beta; IL‐6, interleukin‐6; IL‐8, interleukin‐8; IL‐13, interleukin‐13; MMP3, matrix metalloproteinase 3; MMP‐13, matrix metalloproteinase 13; NITEGE, aggrecan neopeptide; OPG, osteoprotegerin; PPARγ, peroxisome proliferator‐activated receptor‐gamma; RANKL, receptor activator of nuclear factor‐kB ligand.

Biomarkers were classified into five categories based on their clinical utility (Supplementary Materials S5–S7). From a diagnostic perspective, 19 biomarkers were identified. Among these, 10 biomarkers were significantly associated with diagnosis only, as summarised in Table 2 [7, 18, 21, 26, 40]. Forty‐two biomarkers predicting the association of FAIS with hip OA or late FAIS were identified, of which only 14 biomarkers were significantly related only to disease progression (Table 3) [1, 12, 15, 21, 23, 26, 28, 40]. Collagen, type 2, alpha (COL2A1), COL2, DNMT3B, IL‐1b, MMP‐13, Vascular endothelial growth factor (VEGF) and ADAMTS‐4 were controversial among studies (Opinions were divided) (Supplementary Material S8) [12, 15, 21, 23, 26, 28, 40]. 4‐aminobutyrate aminotransferase promoter (ABAT) and Peroxisome proliferator‐activated receptor‐gamma (PPARγ) were related to ‘diagnosis’ and ‘disease progression’ (ABAT: early FAIS vs. nondiseased: *p* < 0.0001, early FAIS vs. late FAIS: *p* = 0.0345; PPARγ: early FAIS vs. nondiseased: *p* = 0.0096, early FAIS vs. late FAIS: *p* = 0.027) (Tables 2 and 3) [26, 40]. The details are shown in Figure 2 and Supplementary Material S8. A detailed summary of statistical comparisons, including *p*‐values and directionality of biomarker differences between study and control groups, is provided in the Supplementary Material.

**Table 2:** Biomarkers for the diagnosis of FAI compared to healthy control.

Marker	Authors	Year	Statistical association	Patient	Control	Data sources
NITEGE	Haneda et al.	2020	Early FAI and late FAI > control	Early FAI	Healthy young adults	Synovial fluid samples
COL10A1	Kamenaga et al.	2023	Positive association with progression of OA	Early and late FAI	Healthy cadavers	Cartilage samples
IL‐6	Gao et al.	2021	Early stage cam‐type FAI > control	Cam‐type FAI	Femoral neck fracture	Bone tissue samples from head–neck junction
ALP	Gao et al.	2021	Early stage cam‐type FAI > control	Cam‐type FAI	Femoral neck fracture	Bone tissue samples from head–neck junction
RANKL	Gao et al.	2021	Early stage cam‐type FAI > control	Cam‐type FAI	Femoral neck fracture	Bone tissue samples from head–neck junction
OPG	Gao et al.	2021	Early stage cam‐type FAI > control	Cam‐type FAI	Femoral neck fracture	Bone tissue samples from head–neck junction
COMP	Bedi et al.	2013	24% increase in FAI compared with controls	FAI	Normal hips	Plasma samples
CRP	Bedi et al.	2013	276% increase in FAI compared with controls	FAI	Normal hips	Plasma samples
DNMT1	Pascual‐Garrido	2022	Negative association with progression of OA	Early FAI	ND	Articular cartilage samples
DNMT3A	Pascual‐Garrido	2022	Negative association with progression of OA	Early FAI	ND	Articular cartilage samples
ABAT	Kamenaga et al.	2023	Positive association with progression of OA	Early and late FAI	Healthy cadavers	Cartilage samples
PPARγ	Pascual‐Garrido	2022	Negative association with progression of OA	Early FAI	ND	Articular cartilage samples

*Note*: 4‐aminobutyrate aminotransferase promoter (ABAT) and peroxisome proliferator‐activated receptor‐gamma (PPARγ) were related to both ‘diagnosis’ and ‘disease progression.’

Abbreviations: FAI, femoroacetabular impingement; ND, non disease; OA, osteoarthritis.

**Table 3:** Biomarkers of ‘disease progression group’ compared to OA.

Marker	Authors	Year	Statistical association	Patient	Control	Data sources
FGF18	Kuhns et al.	2022	343.1‐fold increase compared to OA	FAI	OA due to FAI	Articular cartilage samples
WNT16	Kuhns et al.	2022	57.8‐fold increase compared to OA	FAI	OA due to FAI	Articular cartilage samples
CD68 macrophages	Elias Jones et al.	2015	FAI > OA	FAI	OA	labrum from impingement zone of the lesion
CD34	Elias Jones et al.	2015	FAI > OA	FAI	OA	Labrum from impingement zone of the lesion
IL‐13	Elias Jones et al.	2015	FAI > OA	FAI	OA	Labrum from impingement zone of the lesion
Mast cells	Elias Jones et al.	2015	FAI > OA	FAI	OA	Labrum from impingement zone of the lesion
FAC	Abrams et al.	2014	Non‐OA (1.153) > OA (0.083)	Non‐OA	OA	Synovial fluid samples
IL‐8	Hashimoto	2013	FAI > OA	Non‐OA	OA	Synovial fluid samples
	Chinzei et al.	2016	OA > FAI in synovium	FAI	OA	Cartilage, synovium and labrum
CCL3L1	Hashimoto	2013	FAI > OA	FAI	OA	Articular cartilage samples
ACAN	Hashimoto	2013	FAI > OA	FAI	OA	Articular cartilage samples
	Chinzei et al.	2016	OA > FAI in cartilage	FAI	OA	Cartilage, synovium and labrum
AKT1	Pascual‐Garrido	2022	Early FAI > Late FAI	Early FAI	Late FAI	Articular cartilage samples
HIFα	Pascual‐Garrido	2022	Early FAI < Late FAI	Early FAI	Late FAI	Articular cartilage samples
MMP3	Chinzei et al.	2016	OA > FAI in synovium and labrum	FAI	OA	Cartilage, synovium and labrum
COL1A1	Chinzei et al.	2016	OA > FAI in labrum	FAI	OA	Cartilage, synovium and labrum
ABAT	Kamenaga et al.	2023	Positive association with progression of OA	Early FAI	Late FAI	Articular cartilage samples
PPARγ	Pascual‐Garrido	2022	Early FAI > Late FAI	Early FAI	Late FAI	Articular cartilage samples

*Note*: 4‐aminobutyrate aminotransferase promoter (ABAT) and Peroxisome proliferator‐activated receptor‐gamma (PPARγ) were related to both ‘diagnosis’ and ‘disease progression’.

Abbreviations: DDH, developmental dysplasia of hip; FAI, femoroacetabular impingement; GAPDH, glyceraldehyde 3‐phosphate dehydrogenase; ND, non disease; OA, osteoarthritis.

Two cohort studies investigated 21 biomarkers without control groups. Inflammatory markers such as IL‐1β, IL‐6, TNF‐α and MMP1 increased with synovitis or severe chondral damage (Supplementary Material S7) [16, 48]. Patients with cam deformity showed lower levels of bone turnover markers (e.g., CTX‐I, NTX‐I) and higher serum COMP and HA. However, other markers showed no consistent association [48].

## DISCUSSION

This systematic review identified 12 biomarkers that showed potential diagnostic value for early detection of FAIS, which may support earlier intervention and improved patient outcomes. In addition, 16 biomarkers were associated with disease progression and may have prognostic implications. However, these findings should be interpreted with caution due to heterogeneity in study design, control groups and biomarker measurement methods. Notably, ABAT and PPARγ, both obtained from cartilage, demonstrated both diagnostic and prognostic utility, while serum COMP showed potential for diagnostic screening. Compared to previous reviews, we identified 25 additional biomarkers and reclassified others more precisely; for example, FAC, previously considered a diagnostic marker, was reclassified as prognostic based on updated evidence [33]. These findings offer a foundation for developing biomarker panels and guiding future translational research.

ABAT and PPARγ were related to both the diagnosis and disease progression [26, 40]. ABAT is an enzyme that converts γ‐aminobutyric acid into succinate, which is an essential intermediate in the tricarboxylic acid cycle [46]. Some have proposed that ABAT as a novel target for disease modifying osteoarthritis drugs [26]. Kamenaga et al. reported that IL‐1β‐induced inflammation further reduced DNA methylation at the ABAT promoter, leading to excessive ABAT expression and increased catabolic markers in hip OA chondrocytes, suggesting that this mechanism may accelerate FAIS progression in both early and late stages under persistent inflammatory conditions [26]. PPARγ, expressed in chondrocytes, is crucial for maintaining cartilage homoeostasis [49]. Current progress in OA research suggests that the development of OA is linked to abnormal epigenetic modifications in many genes that make individuals susceptible to OA [44, 52]. Indeed, in knee OA, PPARγ is inhibited, and this inhibition is largely due to the abnormally induced DNMT1/DNMT3 [53]. In the hip, PPARγ, DNMT1 and DNMT3 are significantly elevated in early FAIS compared to healthy controls, and levels increase with disease progression [40].

An ideal biomarker would be collected noninvasively. Indeed, ABAT, PPARγ, DNMT1, DNMT3A, IL‐6, COL10A1, ALP, OPG and RANKL are cartilage/bone samples, and therefore, are collected invasively, such as during arthroscopy. However, COMP and CRP can be collected from serum. COMP, an extracellular matrix protein that organises collagen II matrices [19, 42], has diagnostic value in FAIS [33]. Although CRP can be collected from serum, the clinical utility in FAIS is restricted due to the lack of specificity [38]. At present, it is believed that utilising most of these ‘diagnostic biomarkers’ for routine clinical use is challenging. Nevertheless, these biomarkers further illustrate the notion that FAIS is not simply a mechanical pathology, but one with inflammatory and catabolic components.

Since FAIS progression is associated with poor outcomes after hip arthroscopy [29], identifying early disease and predicting disease progression is vital. Although preoperative MRI has been used to evaluate early articular damage [3, 27, 30, 47], concerns have been raised about sensitivity [13, 29]. Therefore, the clinical use of biomarkers that more sensitively identify cartilage degeneration may be useful in refining surgical indications based on disease stratification. In the disease progression group, 16 biomarkers were shown to be statistically significant. In the group, IL‐8 and ACAN were reported by two studies [12, 23], and other biomarkers were reported by a single study. MMP‐13 and ADAMTS‐4 have been reported to be associated with FAIS in multiple studies, but due to interstudy heterogeneity, they were not included in this group [12, 21, 23, 26, 28, 40]. Moreover, there are many reports that ADAMTS‐4, except for one study, showed a significant difference between early FAIS and OA, and it may be useful as a diagnostic biomarker [12, 21, 23, 28].

Although this systematic review has been performed with robust methodology, there are several limitations. First, the inclusion of only English language studies and those after 1999 may introduce bias. Moreover, the poor quality of evidence, mainly at levels III and IV, makes the overall level of evidence similarly low. In addition, with limited availability of quantitative evaluations for most biomarkers and substantial study heterogeneity, meta‐analysis was not possible. One broader limitation is the sensitivity and accuracy of biomarker measurement—there is a limited amount of synovial fluid to sample, which can be difficult to access and lavage may be required [1]. Despite these limitations, this study aggregated the latest biomarkers in FAIS and identified several biomarkers that may be clinically useful by qualitatively integrating heterogeneous studies. A future challenge is the need for studies with clear and consistent study designs that unify the subject and control groups to diagnose or predict the progression to OA.

Additionally, the generalisability of our findings remains limited by the sample population of included studies, which primarily involved patients undergoing hip arthroscopy. Therefore, the applicability of these biomarkers to asymptomatic individuals or nonoperative FAIS cases remains to be determined. Nevertheless, this systematic review highlights the potential clinical applicability of biomarkers in the diagnosis and prognostication of FAIS. Among the biomarkers identified, serum COMP appears to be the most clinically accessible and promising at present. While immediate clinical implementation remains limited, this study provides an updated classification of biomarkers, distinguishing those with diagnostic versus prognostic utility. These findings may serve as a foundation for future translational research aimed at developing minimally invasive diagnostic tools and refining patient stratification for early intervention.

## CONCLUSION

In FAIS, several biomarkers may be used for identifying early changes, including ABAT, PPARγ, DNMT1, DNMT3A, IL‐6, COL10A1, NITEGE, ALP, OPG, RANKL, COMP and CRP. Moreover, 16 biomarkers were significant predictors of FAIS progression. ABAT and PPARγ were associated with diagnosis and disease progression. These findings may improve the early detection of FAIS, potentially allowing for earlier intervention and more accurate patient stratification. Incorporating molecular biomarkers into clinical evaluation may enhance decision‐making, particularly in ambiguous cases where imaging alone is inconclusive. Future studies with appropriate strategies and proper control groups are necessary to determine which biomarkers will meet the criteria for routine clinical use.

## AUTHOR CONTRIBUTIONS

**Junya Yoshitani**: Conceptualisation; data curation; investigation; writing—original draft; writing—review and editing. **Seper Ekhtiari**: Data curation; writing—review and editing. **Joseph Dulleston**: Writing—review and editing. **Ajay Malviya**: Writing—review and editing. **Vikas Khanduja**: Conceptualisation; reviewer; supervision; writing—review and editing, overall responsibility.

## CONFLICT OF INTEREST STATEMENT

Vikas Khanduja is a consultant to Smith and Nephew and Arthrex. The remaining authors declare no conflict of interest.

## ETHICS STATEMENT

The protocol for the review has been published in PROSPERO (Reg no: CRD42023408097).

## Supporting information

Supporting information.

Supporting information.

## Data Availability

The authors have nothing to report.
