# Supplementary material for: Diagnostic and prognostic potential of biomarkers in femoroacetabular impingement syndrome: A systematic review
Source: J Exp Orthop. 2025 Sep 22;12(3):e70417. doi: 10.1002/jeo2.70417 (PMC12451476; doi:10.1002/jeo2.70417)
Supplement: Supplementary file 1 — Supporting information. [file JEO2-12-e70417-s002.docx]

| Supplementary material 1: Searching strategy | | |
| --- | --- | --- |
| PubMed |  |  |
| Query | Search Details | Results |
| ((marker[tiab]) OR (markers[tiab]) OR (biomarker[tiab]) OR (biomarkers[tiab])) AND (("cam morphology"[tiab]) OR ("cam deformity"[tiab]) OR ("pincer morphology"[tiab]) OR ("pincer deformity"[tiab]) OR ("femoroacetabular impingement"[tiab]) OR ("femoro-acetabular impingement"[tiab]) OR (FAI[tiab]) OR ("hip osteoarthritis"[tiab])) | ("marker"[Title/Abstract] OR "markers"[Title/Abstract] OR "biomarker"[Title/Abstract] OR "biomarkers"[Title/Abstract]) AND ("cam morphology"[Title/Abstract] OR "cam deformity"[Title/Abstract] OR "pincer morphology"[Title/Abstract] OR "pincer deformity"[Title/Abstract] OR "femoroacetabular impingement"[Title/Abstract] OR "femoro-acetabular impingement"[Title/Abstract] OR "FAI"[Title/Abstract] OR "hip osteoarthritis"[Title/Abstract]) | 388 |
| Cochrane review | |  |
| Items | Searching strategy | Results |
| #1 | femoroacetabular impingement | 196 |
| #2 | FAI | 565 |
| #3 | cam morphology | 42 |
| #4 | cam deformity | 32 |
| #5 | pincer morphology | 7 |
| #6 | pincer deformity | 5 |
| #7 | hip osteoarthritis | 4555 |
| #8 | #1 OR #2 OR #3 OR #4 OR #5 OR #6 OR #7 | 5212 |
| #9 | biomarker* | 52375 |
| #10 | #8 AND #9 | 119 |
| Embase <1974 to 2023 May 01> | | |
| Items | Searching strategy | Results |
| 1 | exp femoroacetabular impingement/ | 4790 |
| 2 | exp hip osteoarthritis/ | 14118 |
| 3 | 1 or 2 | 18352 |
| 4 | exp biological marker/ | 440029 |
| 5 | 3 and 4 | 170 |

| Supplementary material 2: Summary of the study characteristics (continued) | | | | | |  |  |  |  |  |  |  |  |  |  |  |
| --- | --- | --- | --- | --- | --- | --- | --- | --- | --- | --- | --- | --- | --- | --- | --- | --- |
| Author | Year | Patients’ diagnosis | Number | Male | Female | MA | Control Diagnosis 1 | Number | Male | Female | MA | Control Diagnosis 2 | Number | Male | Female | MA |
| van Spil et al | 2015 | Knee or hip pain cohort and cam type FAI (alpha angle 60 and clinical sign) | 1583 | 325 | 1258 | 55.8 |  |  |  |  |  |  |  |  |  |  |
| Shapiro et al | 2016 | FAI; Signs and symptoms of hip pain as well as imaging results consistent with FAI | 16 | 4 | 12 | 38.9 |  |  |  |  |  |  |  |  |  |  |
| Haneda et al | 2020 | Early FAI; Hip pain >3 months; FADIR test; and radiographic and intraoperative findings | 15 | 11 | 4 | 32.5 | Late FAI; Tönnis grades 2–3. | 15 | 13 | 2 | 57.1 | Healthy young adult donors | 7 | 2 | 5 | 15.1 |
| Bedi et al | 2013 | FAI; Symptomatic physically active male athletes, 18 to 40 years of age, with radiographically confirmed FAI lesions, and positive anterior impingement and FADIR tests | 10 | 10 | 0 | 23.1 | Physically active male athletes, 18 to 40 years of age, with radiographically normal hips, and negative anterior impingement and FADIR tests | 19 | 19 | 0 | 22.3 |  |  |  |  |  |
| Talks et al | 2019 | FAI confirmed clinically and with imaging performed surgical procedure | 57 |  |  |  | Symptomatic FAI with physiotherapy | 58 |  |  |  |  |  |  |  |  |
| Kamenaga et al | 2023 | Early FAI; Hip pain more than 3 months, hip range of motion, radiographic findings, and intraoperative findings. A cam deformity was defined by an alpha angle greater than 55° on radiographs | 12 | 7 | 5 | 34.5 | Late FAI; Tönnis grades 2–3. | 12 | 5 | 7 | 50.7 | ND healthy samples | 5 | 3 | 2 | 27.7 |
| Kuhns et al | 2022 | FAI alpha angle > 60 | 21 | 13 | 8 | 34.2 | End stage OA secondary to FAI with an alpha angle >60° | 16 | 9 | 7 | 62.7 |  |  |  |  |  |
| Turdean et al | 2017 | Primary hip OA; joint space narrowing and the presence of osteosclerosis and large osteophytes | 16 | 5 | 11 | 67.69 | Rapidly destructive coxopathy; rapid clinical deterioration (in <3 years), the presence of geodes in the acetabulum and femoral head, JSN, flattening and/or partial osteolysis of the femoral head | 24 | 12 | 12 | 67.4 | Avascular necrosis of femoral head; identifying an area of necrosis and collapse of the femoral head | 17 | 15 | 2 | 56.7 |
| Elias Jones et al | 2015 | FAI; Confirmed diagnosis on plain radiographs and/or magnetic resonance imaging and positive clinical examination finding | 10 |  |  | 36 | OA confirmed radiographically and macroscopically | 10 |  |  | 39 |  |  |  |  |  |
| Abrams et al | 2017 | FAI; Hip pain without radiographic evidence of osteoarthritis (Tönnis grade 0-1) as well as a self-reported pain decrease of at least 50% with a diagnostic injection | 12 | 9 | 3 | 34.5 | Instability; Instability is based on some clinical tests and Beighton score of 6 or greater,19 as well as a lack of significant cam or pincer | 5 | 0 | 5 | 30.8 |  |  |  |  |  |
| Abrams et al | 2014 | Non-OA; Tonnis grade 0 | 17 | 5 | 12 | 38.3 | OA; Tonnis grade 2 or greater | 17 | 9 | 8 | 59.2 |  |  |  |  |  |
| Fukushima et al | 2017 | FAI or OA; Labral tears and/or increased synovial fluid identified with pre-operative MRI | 33 | 10 | 23 | 41.8 |  |  |  |  |  |  |  |  |  |  |
| Hashimoto et al | 2013 | FAI; Groin pain, positive impingement test. All were evaluated with preoperative radiographs with cam or combined cam and pincer impingement. | 25 | 17 | 8 | 24.1 | OA; End stage osteoarthritis | 7 | 5 | 2 | 52.7 | DDH | 3 | 0 | 3 | 28 |
| Pascual-Garrido et al | 2022 | Early FAI; Tönnis 0–1 and cam deformity was defined by an alpha angle greater than 55° | 9 | 6 | 3 | 43.5 | Late FAI in patients with Tönnis 2–3. | 13 | 9 | 4 | 59.6 | ND healthy allograft | 5 |  |  |  |
| Gao et al | 2021 | Cam-type FAI; FADIR or FABER tests were considered positive and the alpha angle were calculated | 12 | 4 | 8 | 33.8 | Femoral neck fracture | 6 | 2 | 4 | 55.3 |  |  |  |  |  |
| Chinzei et al | 2016 | FAI; Groin pain, positive impingement test. All were evaluated with preoperative radiographs with cam or combined cam and pincer impingement. | 30 | 18 | 12 | 42 | End stage osteoarthritis | 30 | 2 | 28 | 69 |  |  |  |  |  |

MA: Mean age; FAI: Femoroacetabular impingement; FADIR: Flexion, adduction, internal rotation; FABER: Flexion, abduction and external rotation; ND: Non-disease;

| Supplementally Materials 3: The JBI’s analytical cross-sectional studies critical appraisal tool | | | | | | | | | | | |
| --- | --- | --- | --- | --- | --- | --- | --- | --- | --- | --- | --- |
| Author | Year | Journal | Q1 | Q2 | Q3 | Q4 | Q5 | | Q6 | Q7 | Q8 |
| van Spil et al | 2015 | Osteoarthritis Cartilage | Yes | Yes | Yes | Yes | Yes | | Yes | Yes | Yes |
| Shapiro et al | 2016 | J Hip Preserv Surg | Yes | Yes | Yes | Yes | No | | No | Yes | Yes |
| Haneda et al | 2020 | Am J Sports Med | Yes | Yes | Yes | Yes | Yes | | No | Yes | Yes |
| Bedi et al | 2013 | Am J Sports Med | Yes | Yes | Yes | Yes | Yes | | No | Yes | Yes |
| Talks et al | 2019 | JOR | Yes | No | Yes | Yes | Yes | | Yes | Yes | No |
| Kamenaga | 2023 | JOR | Yes | Yes | Yes | Yes | Yes | | No | Yes | Yes |
| Kuhns et al | 2022 | JOR | Yes | Yes | Yes | Yes | Yes | | No | Yes | Yes |
| Turdean et al | 2017 | J investig Med | Yes | Yes | Yes | Yes | No | | No | Yes | No |
| Elias Jones et al | 2015 | Am J Sports Med | Yes | No | Yes | Yes | Yes | | No | Yes | Yes |
| Abrams et al | 2017 | Arthroscopy | Yes | Yes | Yes | Unclear | Yes | | No | Yes | Yes |
| Abrams et al | 2014 | KSSTA | Yes | Yes | Yes | Yes | No | | No | Yes | Yes |
| Fukushima | 2017 | J Exp Orthop | Yes | Yes | Yes | Yes | No | | No | Yes | Yes |
| Hashimoto | 2013 | J bone Joint Surg Am | Yes | Yes | Yes | Yes | No | | No | Yes | Yes |
| Pascual-Garrido | 2022 | J Arthroplasty | Yes | Yes | Yes | Yes | Yes | | No | Yes | Yes |
| Gao et al | 2021 | J Orthop Surg Res | Yes | Yes | Yes | Yes | Yes | | No | Yes | Yes |
| Chinzei et al | 2016 | J bone Joint Surg Am | Yes | Yes | Yes | Yes | Yes | | No | Yes | Yes |
| Q1. Were the criteria for inclusion in the sample clearly defined? | | | | | | | |  |  |  |  |
| Q2. Were the study subjects and the setting described in detail? | | | | | | | |  |  |  |  |
| Q3. Was the exposure measured in a valid and reliable way? | | | | | | | |  |  |  |  |
| Q4. Were objective, standard criteria used for measurement of the condition? | | | | | | | |  |  |  |  |
| Q5. Were confounding factors identified? | | | | | | | |  |  |  |  |
| Q6. Were strategies to deal with confounding factors stated? | | | | | | | |  |  |  |  |
| Q7. Were the outcomes measured in a valid and reliable way? | | | | | | | |  |  |  |  |
| Q8. Was appropriate statistical analysis used? | | | | | | | |  |  |  |  |

| Supplementary material 4: Summary of Biomarkers | | |  |  | |  | |  |  | | |  | |  | |  |  | |  | | |  | |  |  | |  | |  | |  | |  | |
| --- | --- | --- | --- | --- | --- | --- | --- | --- | --- | --- | --- | --- | --- | --- | --- | --- | --- | --- | --- | --- | --- | --- | --- | --- | --- | --- | --- | --- | --- | --- | --- | --- | --- | --- |
| Marker | Abbreviation | Classification | | Hashimoto et al. | Bedi et al. | | Abrams et al. | | | Elias Jones et al. | van Spil et al. | | Sharpio et al. | | Chinzei et al | | | Abrams et al. | | Fukushima et al. | Turdean et al. | | Talks et al. | | | Haneda et al. | | Gao et al | | Kuhn et al. | | Pascual-Garrido et al. | | Kamenaga et al. |
|  |  |  |  | 2013 | 2013 | | 2014 | | | 2015 | 2015 | | 2016 | | 2016 | | | 2017 | | 2017 | 2017 | | 2019 | | | 2020 | | 2021 | | 2022 | | 2022 | | 2023 |
| IL-1 | Interleukin-1 beta | Biomarkers related to other processes | Cytokines |  |  | |  | | |  |  | |  | |  | | |  | |  |  | |  | | |  | | No | |  | |  | |  |
| IL-1b | Interleukin-1 beta | Biomarkers related to other processes | Cytokines | No |  | |  | | |  |  | | No | | ✓ | | |  | | ✓ |  | |  | | | ✓ | |  | |  | |  | |  |
| IL-6 | Interleukin-6 | Biomarkers related to other processes | Cytokines |  |  | | No | | |  |  | | No | |  | | |  | | ✓ |  | |  | | |  | | ✓ | |  | |  | |  |
| IL-8 | Interleukin-8 | Biomarkers related to other processes | Cytokines | ✓ |  | |  | | |  |  | |  | | ✓ | | |  | |  |  | |  | | |  | | No | |  | |  | |  |
| IL-13 | Interleukin-13 | Biomarkers related to other processes | Cytokines |  |  | |  | | | ✓ |  | |  | |  | | |  | |  |  | |  | | |  | |  | |  | |  | |  |
| IL-1RA | Interleukin-1 receptor agonist | Biomarkers related to other processes | Cytokines |  |  | | No | | |  |  | | No | |  | | |  | |  |  | |  | | |  | |  | |  | |  | |  |
| IFN-γ | Interferon γ | Biomarkers related to other processes | Cytokines |  |  | | No | | |  |  | | No | |  | | |  | |  |  | |  | | |  | |  | |  | |  | |  |
| TNFα | Tumor necrosis factor-alpha | Biomarkers related to other processes | Cytokines |  |  | | No | | |  |  | | No | |  | | |  | | ✓ |  | |  | | |  | |  | |  | |  | |  |
| CXCL1 | Chemokine (C-X-C) motif ligand 1 | Biomarkers related to other processes | Chemokines | No |  | |  | | |  |  | |  | |  | | |  | |  |  | |  | | |  | |  | |  | |  | |  |
| CXCL2 | Chemokine (C-X-C) motif ligand 2 | Biomarkers related to other processes | Chemokines | No |  | |  | | |  |  | |  | |  | | |  | |  |  | |  | | |  | |  | |  | |  | |  |
| CXCL3 | Chemokine (C-X-C) motif ligand 3 | Biomarkers related to other processes | Chemokines | No |  | |  | | |  |  | |  | |  | | |  | |  |  | |  | | |  | |  | |  | |  | |  |
| CXCL6 | Chemokine (C-X-C) motif ligand 6 | Biomarkers related to other processes | Chemokines | No |  | |  | | |  |  | |  | |  | | |  | |  |  | |  | | |  | |  | |  | |  | |  |
| CCL3 | Chemokine (C-C) motif ligand 3 | Biomarkers related to other processes | Chemokines | No |  | |  | | |  |  | | No | |  | | |  | |  |  | |  | | |  | |  | |  | |  | |  |
| CCL3L1 | Chemokine (C-C) motif ligand 3-like 1 | Biomarkers related to other processes | Chemokines | ✓ |  | |  | | |  |  | |  | |  | | |  | |  |  | |  | | |  | |  | |  | |  | |  |
| MCP-1 | Monocyte chemoattractant protein-1 | Biomarkers related to other processes | Chemokines |  |  | | No | | |  |  | | No | |  | | |  | |  |  | |  | | |  | |  | |  | |  | |  |
| Eotaxin |  | Biomarkers related to other processes | Chemokines |  |  | | No | | |  |  | | No | |  | | |  | |  |  | |  | | |  | |  | |  | |  | |  |
| IP-10 CXCL10 | Interferon gamma induced protein 10 | Biomarkers related to other processes | Chemokines |  |  | | No | | |  |  | | No | |  | | |  | |  |  | |  | | |  | |  | |  | |  | |  |
| RANTES CCL5 | Regulated on activation, normal T cell expressed and secreted | Biomarkers related to other processes | Chemokines |  |  | | No | | |  |  | | No | |  | | |  | |  |  | |  | | |  | |  | |  | |  | |  |
| MMP1 | Matrix metalloproteinase | Biomarkers related to other non-collagenous proteins | Matrix metalloproteinases |  |  | |  | | |  |  | |  | |  | | |  | | ✓ |  | |  | | |  | |  | |  | |  | |  |
| MMP3 | Matrix metalloproteinase | Biomarkers related to other non-collagenous proteins | Matrix metalloproteinases |  |  | |  | | |  |  | |  | | ✓ | | |  | | No |  | |  | | |  | |  | |  | |  | |  |
| MMP-13 | Matrix metalloproteinase 13 | Biomarkers related to other non-collagenous proteins | Matrix metalloproteinases | No |  | |  | | |  |  | |  | | ✓ | | |  | |  |  | |  | | | ✓ | |  | | ✓ | | ✓ | | ✓ |
| COMP | Cartilage oligomeric matrix protein | Biomarkers related to other non-collagenous proteins |  |  | ✓ | |  | | |  | ✓* | |  | |  | | |  | |  |  | |  | | |  | |  | |  | |  | |  |
| OC | Osteocalcin | Biomarkers related to other non-collagenous proteins |  |  |  | |  | | |  | ✓ | |  | |  | | |  | |  |  | |  | | |  | |  | |  | |  | |  |
| HA | Hyaluronic acid | Biomarkers related to other non-collagenous proteins |  |  |  | |  | | |  | ✓* | |  | |  | | |  | |  |  | |  | | |  | |  | |  | |  | |  |
| ADAMTS-4 | A disintegrin and metalloproteinase with thrombospondin motifs 4 | Biomarkers related to aggrecan metabolism |  | ✓ |  | |  | | |  |  | |  | | ✓ | | |  | | ✓ |  | |  | | | ✓ | |  | | ✓ | |  | |  |
| ACAN | Aggrecan | Biomarkers related to aggrecan metabolism |  | ✓ |  | |  | | |  |  | |  | | ✓ | | |  | |  |  | |  | | |  | |  | |  | |  | |  |
| NITEGE | Aggrecan neopeptide | Biomarkers related to aggrecan metabolism |  |  |  | |  | | |  |  | |  | |  | | |  | |  |  | |  | | | ✓ | |  | |  | |  | |  |
| FAC | Fibronectin–aggrecan complex | Biomarkers related to aggrecan metabolism |  |  |  | | ✓ | | |  |  | | No | |  | | |  | |  |  | |  | | |  | |  | |  | |  | |  |
| sCS846 | Chondroitin sulphate 846 | Biomarkers related to aggrecan metabolism |  |  |  | |  | | |  | No | |  | |  | | |  | |  |  | |  | | |  | |  | |  | |  | |  |
| COL2A1, COL2 | Collagen, type 2, alpha | Biomarkers related to collagen metabolism |  | ✓ |  | |  | | |  |  | |  | | ✓ | | |  | |  |  | |  | | | No | |  | |  | | No | | No |
| COL1A1 | Collagen, type 1 alpha 1 | Biomarkers related to collagen metabolism |  |  |  | |  | | |  |  | |  | | ✓ | | |  | |  |  | |  | | |  | |  | |  | |  | |  |
| COL10A1 | Collagen Type X Alpha 1 Chain | Biomarkers related to collagen metabolism |  |  |  | |  | | |  |  | |  | |  | | |  | |  |  | |  | | |  | |  | |  | |  | | ✓ |
| uCTX-I | C-terminal telopeptide of collagen type I | Biomarkers related to collagen metabolism |  |  |  | |  | | |  | ✓* | |  | |  | | |  | |  |  | |  | | |  | |  | |  | |  | |  |
| uNTX-I | N-terminal telopeptide of collagen type I | Biomarkers related to collagen metabolism |  |  |  | |  | | |  | ✓* | |  | |  | | |  | |  |  | |  | | |  | |  | |  | |  | |  |
| sPINP | N-terminal propeptide of procollagen type I | Biomarkers related to collagen metabolism |  |  |  | |  | | |  | ✓* | |  | |  | | |  | |  |  | |  | | |  | |  | |  | |  | |  |
| uCTX-Ⅱ | C-terminal telopeptide of collagen type II | Biomarkers related to collagen metabolism |  |  |  | |  | | |  | No | |  | |  | | |  | |  |  | |  | | |  | |  | |  | |  | |  |
| sPIIANP | N-terminal propeptide of procollagen type IIA | Biomarkers related to collagen metabolism |  |  |  | |  | | |  | ✓* | |  | |  | | |  | |  |  | |  | | |  | |  | |  | |  | |  |
| sPIIINP | N-terminal propeptide of procollagen type III | Biomarkers related to collagen metabolism |  |  |  | |  | | |  | No | |  | |  | | |  | |  |  | |  | | |  | |  | |  | |  | |  |
| FGF18 | Fibroblast growth factor | Biomarkers related to other processes | Growth factors |  |  | |  | | |  |  | |  | |  | | |  | |  |  | |  | | |  | |  | | ✓ | |  | |  |
| VEGF | Vascular endothelial growth factor | Biomarkers related to other processes | Growth factors |  |  | | No | | | ✓ |  | | No | |  | | |  | |  |  | |  | | |  | |  | |  | |  | |  |
| PDGF-BB | Platelet-derived growth factor | Biomarkers related to other processes | Growth factors |  |  | | No | | |  |  | | No | |  | | |  | |  |  | |  | | |  | |  | |  | |  | |  |
| pLeptin |  | Biomarkers related to other processes | Adipokines |  |  | |  | | |  | ✓* | |  | |  | | |  | |  |  | |  | | |  | |  | |  | |  | |  |
| pAdiponectin |  | Biomarkers related to other processes | Adipokines |  |  | |  | | |  | ✓* | |  | |  | | |  | |  |  | |  | | |  | |  | |  | |  | |  |
| pResistin |  | Biomarkers related to other processes | Adipokines |  |  | |  | | |  | No | |  | |  | | |  | |  |  | |  | | |  | |  | |  | |  | |  |
| ALP | Alkaline phosphatase | Biomarkers related to other processes | Bone or cartilage metabolism |  |  | |  | | |  |  | |  | |  | | |  | |  |  | |  | | |  | | ✓ | |  | |  | |  |
| OPG | Osteoprotegerin | Biomarkers related to other processes | Bone or cartilage metabolism |  |  | |  | | |  |  | |  | |  | | |  | |  |  | |  | | |  | | ✓ | |  | |  | |  |
| WNT16 |  | Biomarkers related to other processes | Others |  |  | |  | | |  |  | |  | |  | | |  | |  |  | |  | | |  | |  | | ✓ | |  | |  |
| CD31/CD45/CD68 | Cluster of differentiation | Biomarkers related to other processes | Others |  |  | |  | | | ✓ |  | |  | |  | | | No | |  |  | |  | | |  | |  | |  | |  | |  |
| CD3 |  | Biomarkers related to other processes | Others |  |  | |  | | | No |  | |  | |  | | |  | |  |  | |  | | |  | |  | |  | |  | |  |
| CD4 |  | Biomarkers related to other processes | Others |  |  | |  | | | No |  | |  | |  | | |  | |  |  | |  | | |  | |  | |  | |  | |  |
| CD34 |  | Biomarkers related to other processes | Others |  |  | |  | | | ✓ |  | |  | |  | | |  | |  |  | |  | | |  | |  | |  | |  | |  |
| CD206 |  | Biomarkers related to other processes | Others |  |  | |  | | | No |  | |  | |  | | |  | |  |  | |  | | |  | |  | |  | |  | |  |
| Mast cells |  | Biomarkers related to other processes | Others |  |  | |  | | | ✓ |  | |  | |  | | |  | |  |  | |  | | |  | |  | |  | |  | |  |
| CD44/CD105 |  | Biomarkers related to other processes | Others |  |  | |  | | |  |  | |  | |  | | |  | |  | No | |  | | |  | |  | |  | |  | |  |
| DNMT1 | DNA Methyltransferases | Biomarkers related to other processes | Others |  |  | |  | | |  |  | |  | |  | | |  | |  |  | |  | | |  | |  | |  | | ✓ | |  |
| DNMT3A | DNA Methyltransferases | Biomarkers related to other processes | Others |  |  | |  | | |  |  | |  | |  | | |  | |  |  | |  | | |  | |  | |  | | ✓ | |  |
| DNMT3B | DNA methyltransferase 3B | Biomarkers related to other processes | Others |  |  | |  | | |  |  | |  | |  | | |  | |  |  | |  | | |  | |  | |  | | ✓ | | ✓ |
| ABAT | 4‐aminobutyrate aminotransferase promoter | Biomarkers related to other processes | Others |  |  | |  | | |  |  | |  | |  | | |  | |  |  | |  | | |  | |  | |  | |  | | ✓ |
| CRP, hsCRP | C-reactive protein | Biomarkers related to other processes | Others |  | ✓ | |  | | |  | No | |  | |  | | |  | |  |  | |  | | |  | |  | |  | |  | |  |
| κsFLC | Serum-free light chains | Biomarkers related to other processes | Others |  |  | |  | | |  |  | |  | |  | | |  | |  |  | | No | | |  | |  | |  | |  | |  |
| λsFLC |  | Biomarkers related to other processes | Others |  |  | |  | | |  |  | |  | |  | | |  | |  |  | | No | | |  | |  | |  | |  | |  |
| polyclonal SFC |  | Biomarkers related to other processes | Others |  |  | |  | | |  |  | |  | |  | | |  | |  |  | | No | | |  | |  | |  | |  | |  |
| κλratio |  | Biomarkers related to other processes | Others |  |  | |  | | |  |  | |  | |  | | |  | |  |  | | No | | |  | |  | |  | |  | |  |
| ESR | Erythrocyte sedimentation rate | Biomarkers related to other processes | Others |  |  | |  | | |  | ✓* | |  | |  | | |  | |  |  | |  | | |  | |  | |  | |  | |  |
| PPARγ | Peroxisome proliferator-activated receptor-gamma | Biomarkers related to other processes | Others |  |  | |  | | |  |  | |  | |  | | |  | |  |  | |  | | |  | |  | |  | | ✓ | |  |
| AKT1 | Not detected in the original paper | Biomarkers related to other processes | Others |  |  | |  | | |  |  | |  | |  | | |  | |  |  | |  | | |  | |  | |  | | ✓ | |  |
| HIFα | Not detected in the original paper | Biomarkers related to other processes | Others |  |  | |  | | |  |  | |  | |  | | |  | |  |  | |  | | |  | |  | |  | | ✓ | |  |
| RANKL | Receptor activator of nuclear factor-kB ligand | Biomarkers related to other processes | Others |  |  | |  | | |  |  | |  | |  | | |  | |  |  | |  | | |  | | ✓ | |  | |  | |  |

Check marks show that the biomarker showed a significant difference compared to some of the control groups in the study, No indicating that the biomarker did not show any significant difference compared to the controls in the study; check stars show that the biomarker showed a significant difference before adjusting for some demographics such as sex, but after adjusting for them, there was no significant difference.

| Supplementary Material 5: Biomarkers for the diagnosis of FAI compared to healthy control | | | | | | | | |
| --- | --- | --- | --- | --- | --- | --- | --- | --- |
| Marker | Authors | Year | Value | Significant difference | Statistical association | Patient | Control | Data sources |
| IL-1b | Haneda et al | 2020 | % of immunopositive cells | Yes (p < 0.01) | Early FAI and late FAI > control | Early FAI | Healthy young adults | Synovial fluid samples |
| IL-1 | Gao et al | 2021 | Relative gene expression | No | No significant difference | Cam-type FAI | Femoral neck fracture | Bone tissue samples from head-neck junction |
| MMP-13 | Haneda et al | 2020 | % of immunopositive cells | Yes (p = 0.012) | Early FAI and late FAI > control | Early FAI | Healthy young adults | Synovial fluid samples |
|  | Kamenaga et al | 2023 | Relative expression, fold change | Yes (Early FAI vs ND: p = 0.004, Late FAI vs ND: p = 0.0002) | Positive association with progression of OA | Early and late FAI | Healthy cadavers | Cartilage samples |
|  | Pascual-Garrido | 2022 | Relative expression, fold change | Yes (Early FAI vs ND: p = 0.0033, Late FAI vs ND: p = 0.0005) | Positive association with progression of OA | Early FAI | ND | Articular cartilage samples |
| ADAMTS-4 | Haneda et al | 2020 | % of immunopositive cells | Yes (p < 0.01) | Early FAI and late FAI > control | Early FAI | Healthy young adults | Synovial fluid samples |
| NITEGE | Haneda et al | 2020 | % of immunopositive cells | Yes (p < 0.01) | Early FAI and late FAI > control | Early FAI | Healthy young adults | Synovial fluid samples |
| COL2A1, COL2 | Haneda et al | 2020 | % of immunopositive cells | No | No significant difference | Early FAI | Healthy young adults | Synovial fluid samples |
|  | Kamenaga et al | 2023 | Relative expression, fold change | No | No significant difference | Early and late FAI | Healthy cadavers | Cartilage samples |
|  | Pascual-Garrido | 2022 | Relative expression, fold change | No | No significant difference between ND and FAI | Early FAI | ND | Articular cartilage samples |
| DNMT3B | Kamenaga et al | 2023 | Relative expression, fold change | Yes (Early FAI vs ND: p = 0.0006, Late FAI vs ND: p < 0.0001) | Negative association with progression of OA | Early and late FAI | Healthy cadavers | Cartilage samples |
|  | Pascual-Garrido | 2022 | Relative expression, fold change | Yes (Early FAI vs ND: p = 0.0003, Late FAI vs ND: p < 0.0001) | Negative association with progression of OA | Early FAI | ND | Articular cartilage samples |
| ABAT | Kamenaga et al | 2023 | Relative expression, fold change | Yes (Early FAI vs ND: p < 0.0001, Late FAI vs ND: p < 0.0001) | Positive association with progression of OA | Early and late FAI | Healthy cadavers | Cartilage samples |
| COL10A1 | Kamenaga et al | 2023 | Relative expression, fold change | Yes (Early FAI vs ND: p = 0.0003, Late FAI vs ND: p < 0.0001) | Positive association with progression of OA | Early and late FAI | Healthy cadavers | Cartilage samples |
| IL-6 | Gao et al | 2021 | Relative gene expression | Yes (p < 0.05) | Early stage cam-type FAI > control | Cam-type FAI | Femoral neck fracture | Bone tissue samples from head-neck junction |
| IL-8 | Gao et al | 2021 | Relative gene expression | No | No significant difference | Cam-type FAI | Femoral neck fracture | Bone tissue samples from head-neck junction |
| ALP | Gao et al | 2021 | Relative gene expression | Yes (p < 0.05) | Early stage cam-type FAI > control | Cam-type FAI | Femoral neck fracture | Bone tissue samples from head-neck junction |
| RANKL | Gao et al | 2021 | Relative gene expression | Yes (p < 0.05) | Early stage cam-type FAI > control | Cam-type FAI | Femoral neck fracture | Bone tissue samples from head-neck junction |
| OPG | Gao et al | 2021 | Relative gene expression | Yes (p < 0.05) | Early stage cam-type FAI > control | Cam-type FAI | Femoral neck fracture | Bone tissue samples from head-neck junction |
| COMP | Bedi et al | 2013 | μg/L | Yes (p < 0.05) | 24% increase in FAI compared with controls | FAI | Normal hips | Plasma samples |
| CRP | Bedi et al | 2013 | mg/L | Yes (p < 0.05) | 276% increase in FAI compared with controls | FAI | Normal hips | Plasma samples |
| PPARγ | Pascual-Garrido | 2022 | Relative expression, fold change | Yes (Early FAI vs ND: p = 0.00096, Late FAI vs ND: p < 0.0001) | Negative association with progression of OA | Early FAI | ND | Articular cartilage samples |
| DNMT1 | Pascual-Garrido | 2022 | Relative expression, fold change | Yes (Early FAI vs ND: p = 0.0005, Late FAI vs ND: p < 0.0001) | Negative association with progression of OA | Early FAI | ND | Articular cartilage samples |
| DNMT3A | Pascual-Garrido | 2022 | Relative expression, fold change | Yes (Early FAI vs ND: p = 0.0059, Late FAI vs ND: p < 0.0001) | Negative association with progression of OA | Early FAI | ND | Articular cartilage samples |

| Supplementary Material 6: Biomarkers of “disease progression group” compared to OA | | | | | | | | |  |
| --- | --- | --- | --- | --- | --- | --- | --- | --- | --- |
| Marker | Authors | Year | Value | Significant difference | Statistical association | Patient | Control | Data sources |  |
| IL-1b | Haneda et al | 2020 | % of immunopositive cells | No | No significant difference | Early FAI | Late FAI | Synovial fluid samples |  |
|  | Hashimoto | 2013 | Normalized mRNA expression to GAPDH | No | No significant difference | FAI | OA | Articular cartilage samples |  |
|  | Chinzei et al | 2016 | Relative expression of mRNA | Yes (p < 0.01) | OA > FAI in synovium, FAI > OA in cartilage | FAI | OA | Cartilage, synovium and labrum |  |
| MMP-13 | Haneda et al | 2020 | % of immunopositive cells | No | No significant difference | Early FAI | Late FAI | Synovial fluid samples |  |
|  | Kamenaga et al | 2023 | Relative expression, fold change | No (p = 0.2597) | No significant difference | Early FAI | Late FAI | Articular cartilage samples |  |
|  | Kuhns et al | 2022 | Relative fold change to OA | Yes (p < 0.05) | Upregulated in osteoarthritic and high-grade FAI | FAI | OA due to FAI | cartilage samples |  |
|  | Hashimoto | 2013 | Normalized mRNA expression to GAPDH | No | No significant difference | FAI | OA | Articular cartilage samples |  |
|  | Pascual-Garrido | 2022 | Relative expression, fold change | No | No significant difference between early and late FAI | Early FAI | Late FAI | Articular cartilage samples |  |
|  | Chinzei et al | 2016 | Relative expression of mRNA | Yes (p < 0.01) | FAI > OA in cartilage | FAI | OA | Cartilage, synovium and labrum |  |
| ADAMTS-4 | Haneda et al | 2020 | % of immunopositive cells | No | No significant difference | Early FAI | Late FAI | Synovial fluid samples |  |
|  | Kuhns et al | 2022 | Relative fold change to OA | Yes (p < 0.05) | Early FAI < OA | FAI | OA due to FAI | Articular cartilage samples |  |
|  | Hashimoto | 2013 | Normalized mRNA expression to GAPDH | Yes (p < 0.05) | FAI > OA | FAI | OA | Articular cartilage samples |  |
|  | Chinzei et al | 2016 | Relative expression of mRNA | Yes (p < 0.01) | FAI > OA in cartilage | FAI | OA | Cartilage, synovium and labrum |  |
| NITEGE | Haneda et al | 2020 | % of immunopositive cells | No | No significant difference | Early FAI | Late FAI | Synovial fluid samples |  |
| COL2A1, COL2 | Haneda et al | 2020 | % of immunopositive cells | No | No significant difference | Early FAI | Late FAI | Synovial fluid samples |  |
|  | Kamenaga et al | 2023 | Relative expression, fold change | No | No significant difference | Early FAI | Late FAI | Articular cartilage samples |  |
|  | Hashimoto | 2013 | Normalized mRNA expression to GAPDH | No | No significant difference | FAI | OA | Articular cartilage samples |  |
|  | Pascual-Garrido | 2022 | Relative expression, fold change | No | No significant difference between early and late FAI | Early FAI | Late FAI | Articular cartilage samples |  |
|  | Chinzei et al | 2016 | Relative expression of mRNA | Yes (p < 0.01) | OA > FAI in cartilage | FAI | OA | Cartilage, synovium and labrum |  |
| DNMT3B | Kamenaga et al | 2023 | Relative expression, fold change | Yes (p = 0.0164) | Negative association with progression of OA | Early FAI | Late FAI | Articular cartilage samples |  |
|  | Pascual-Garrido | 2022 | Relative expression, fold change | No (p = 0.0603) | No significant difference between early and late FAI | Early FAI | Late FAI | Articular cartilage samples |  |
| ABAT | Kamenaga et al | 2023 | Relative expression, fold change | Yes (p = 0.0345) | Positive association with progression of OA | Early FAI | Late FAI | Articular cartilage samples |  |
| COL10A1 | Kamenaga et al | 2023 | Relative expression, fold change | No (p = 0.5024) | No significant difference between early and late FAI | Early FAI | Late FAI | Articular cartilage samples |  |
| FGF18 | Kuhns et al | 2022 | Relative fold change to OA | Yes (p < 0.05) | 343.1‐fold increase compared to OA | FAI | OA due to FAI | Articular cartilage samples |  |
| WNT16 | Kuhns et al | 2022 | Relative fold change to OA | Yes (p < 0.05) | 57.8‐fold increase compared to OA | FAI | OA due to FAI | Articular cartilage samples |  |
| CD68 macrophages | Elias Jones et al | 2015 | Inflammatory cell score | Yes (p < 0.01) | FAI > OA | FAI | OA | labrum from impingement zone of the lesion | |
| VEGF | Elias Jones et al | 2015 | Inflammatory cell score | Yes (p < 0.01) | FAI > OA | FAI | OA | labrum from impingement zone of the lesion | |
|  | Abrams et al | 2014 | Absolute values | No | No significant difference | non-OA | OA | synovial fluid samples |  |
| CD3 | Elias Jones et al | 2015 | Inflammatory cell score | No | No significant difference | FAI | OA | Labrum from impingement zone of the lesion | |
| CD34 | Elias Jones et al | 2015 | Inflammatory cell score | Yes (p < 0.05) | FAI > OA | FAI | OA | Labrum from impingement zone of the lesion | |
| CD206 | Elias Jones et al | 2015 | Subtype immunostaining | No | No statistical analysis | FAI | OA | Labrum from impingement zone of the lesion | |
| IL-13 | Elias Jones et al | 2015 | Inflammatory cell score | Yes (p < 0.05) | FAI > OA | FAI | OA | Labrum from impingement zone of the lesion | |
| Mast cells | Elias Jones et al | 2015 | Inflammatory cell score | Yes (p < 0.05) | FAI > OA | FAI | OA | Labrum from impingement zone of the lesion | |
| FAC | Abrams et al | 2014 | Optimal density | Yes (p < 0.001) | Non-OA (1.153) > OA (0.083) | Non-OA | OA | Synovial fluid samples |  |
| IFN-γ | Abrams et al | 2014 | pg/ml | No | No significant difference | Non-OA | OA | Synovial fluid samples |  |
| IL-6 | Abrams et al | 2014 | Absolute values | No | No significant difference | Non-OA | OA | Synovial fluid samples |  |
| IL-1RA | Abrams et al | 2014 | Absolute values | No | No significant difference | Non-OA | OA | Synovial fluid samples |  |
| MCP-1 | Abrams et al | 2014 | Absolute values | No | No significant difference | Non-OA | OA | Synovial fluid samples |  |
| Eotaxin | Abrams et al | 2014 | Absolute values | No | No significant difference | Non-OA | OA | Synovial fluid samples |  |
| IP-10 CXCL10 | Abrams et al | 2014 | Absolute values | No | No significant difference | Non-OA | OA | Synovial fluid samples |  |
| PDGF-BB | Abrams et al | 2014 | Absolute values | No | No significant difference | Non-OA | OA | Synovial fluid samples |  |
| RANTES CCL5 | Abrams et al | 2014 | Absolute values | No | No significant difference | Non-OA | OA | Synovial fluid samples |  |
| TNFα | Abrams et al | 2014 | Absolute values | No | No significant difference | Non-OA | OA | Synovial fluid samples |  |
| IL-8 | Hashimoto | 2013 | Normalized mRNA expression to GAPDH | Yes (p < 0.05) | FAI > OA | Non-OA | OA | Synovial fluid samples |  |
|  | Chinzei et al | 2016 | Relative expression of mRNA | Yes (p < 0.01) | OA > FAI in synovium | FAI | OA | Cartilage, synovium and labrum |  |
| CXCL1 | Hashimoto | 2013 | Normalized mRNA expression to GAPDH | No | No significant difference | FAI | OA | Articular cartilage samples |  |
| CXCL2 | Hashimoto | 2013 | Normalized mRNA expression to GAPDH | No | No significant difference | FAI | OA | Articular cartilage samples |  |
| CXCL3 | Hashimoto | 2013 | Normalized mRNA expression to GAPDH | No | No significant difference (vs control p < 0.05) | FAI | OA | Articular cartilage samples |  |
| CXCL6 | Hashimoto | 2013 | Normalized mRNA expression to GAPDH | No | No significant difference (vs control p < 0.05) | FAI | OA | Articular cartilage samples |  |
| CCL3 | Hashimoto | 2013 | Normalized mRNA expression to GAPDH | No | No significant difference | FAI | OA | Articular cartilage samples |  |
| CCL3L1 | Hashimoto | 2013 | Normalized mRNA expression to GAPDH | Yes (p < 0.05) | FAI > OA | FAI | OA | Articular cartilage samples |  |
| ACAN | Hashimoto | 2013 | Normalized mRNA expression to GAPDH | Yes (p < 0.05) | FAI > OA | FAI | OA | Articular cartilage samples |  |
|  | Chinzei et al | 2016 | Relative expression of mRNA | Yes (p < 0.01) | OA > FAI in cartilage | FAI | OA | Cartilage, synovium and labrum |  |
| PPARγ | Pascual-Garrido | 2022 | Relative expression, fold change | Yes (p < 0.05) | Early FAI > Late FAI | Early FAI | Late FAI | Articular cartilage samples |  |
| DNMT1 | Pascual-Garrido | 2022 | Relative expression, fold change | No | No significant difference between early and late FAI | Early FAI | Late FAI | Articular cartilage samples |  |
| DNMT3A | Pascual-Garrido | 2022 | Relative expression, fold change | No | No significant difference between early and late FAI | Early FAI | Late FAI | Articular cartilage samples |  |
| AKT1 | Pascual-Garrido | 2022 | Relative expression, fold change | Yes (p < 0.05) | Early FAI > Late FAI | Early FAI | Late FAI | Articular cartilage samples |  |
| HIFα | Pascual-Garrido | 2022 | Relative expression, fold change | Yes (p < 0.05) | Early FAI < Late FAI | Early FAI | Late FAI | Articular cartilage samples |  |
| MMP3 | Chinzei et al | 2016 | Relative expression of mRNA | Yes (p < 0.01) | OA > FAI in synvium and labrum | FAI | OA | Cartilage, synovium and labrum |  |
| COL1A1 | Chinzei et al | 2016 | Relative expression of mRNA | Yes (p < 0.01) | OA > FAI in labrum | FAI | OA | Cartilage, synovium and labrum |  |

| Supplementary Material 7: Biomarkers in Cohort Study without a control group | | | |  |
| --- | --- | --- | --- | --- |
| Marker | Authors | Year | Statistical association | |
| IL-1b | Fukushima | 2017 | Significant increase in grade 3 synovitis compared to grade 2 patients | |
| IL-6 | Fukushima | 2017 | Significant increase in grade 3 synovitis compared to grade 2 patients | |
| TNFα | Fukushima | 2017 | In Outerbridge grade 4 chondral injury, the levels were significantly increased. Significant increase in grade 3 synovitis compared to grade 2 patients | |
| MMP1 | Fukushima | 2017 | Significant increase in grade 3 synovitis compared to grade 2 patients | |
| sOC | van Spil et al | 2015 | Negative association with cam deformity | |
| sHA | van Spil et al | 2015 | Positive association with cam deformity, but disappeared after adjustment | |
| sPIIANP | van Spil et al | 2015 | Negative interaction with cam deformity in its association with future hip OA | |
| COMP, sCOMP | van Spil et al | 2015 | Positive association with cam deformity, but disappeared after adjustment | |
| sPINP | van Spil et al | 2015 | Negative association with cam deformity, but disappeared after adjustment | |
| uNTX-I | van Spil et al | 2015 | Negative association with cam deformity, but disappeared after adjustment | |
| uCTX-I | van Spil et al | 2015 | Negative association with cam deformity, but disappeared after adjustment | |
| ESR | van Spil et al | 2015 | Negative association with cam deformity, but disappeared after adjustment | |
| pAdiponectin | van Spil et al | 2015 | Negative association with cam deformity, but disappeared after adjustment | |
| pLeptin | van Spil et al | 2015 | Negative association with cam deformity, but disappeared after adjustment | |
| ADAMTS-4 | Fukushima | 2017 | Correlation between hip function or pain | |
| MMP3 | Fukushima | 2017 | No significant difference | |
| sCS846 | van Spil et al | 2015 | No significant difference | |
| uCTX-Ⅱ | van Spil et al | 2015 | No significant difference | |
| sPIIINP | van Spil et al | 2015 | No significant difference | |
| pResistin | van Spil et al | 2015 | No significant difference | |
| CRP, hsCRP | van Spil et al | 2015 | No significant difference | |

| Supplementary material 8: Subgroups of Biomarkers | |  |  |  |
| --- | --- | --- | --- | --- |
| Diagnosis and disease progression | Diagnosis | Disease progression | No significant relation | Opinions were divided |
| ABAT | DNMT1 | IL-8 | CCL3 | COL2A1, COL2 |
| PPARγ | DNMT3A | ACAN | CD206 | VEGF |
|  | IL-6 | AKT1 | CD3 | ADAMTS-4 |
|  | COL10A1 | CCL3L1 | Eotaxin | DNMT3B |
|  | NITEGE | CD34 | IFN-γ | MMP-13 |
|  | ALP | CD68 macrophages | IL-1 | IL-1b |
|  | COMP | COL1A1 | IL-1RA |  |
|  | CRP | CXCL1 | IP-10 CXCL10 |  |
|  | OPG | CXCL2 | MCP-1 |  |
|  |  | CXCL3 | PDGF-BB |  |
|  |  | CXCL6 | RANTES CCL5 |  |
|  |  | FAC | TNFα |  |
|  |  | FGF18 |  |  |
|  |  | HIFα |  |  |
|  |  | IL-13 |  |  |
|  |  | Mast cells |  |  |
|  |  | MMP3 |  |  |
|  |  | RANKL |  |  |
|  |  | WNT16 |  |  |
